# Metamotifs - a generative model for building families of nucleotide position weight matrices

**DOI:** 10.1186/1471-2105-11-348

**Published:** 2010-06-25

**Authors:** Matias Piipari, Thomas A Down, Tim JP Hubbard

**Affiliations:** 1 Wellcome Trust Sanger Institute, Hinxton, Cambridgeshire, UK; 2 Wellcome Trust/Cancer Research UK Gurdon Institute, University of Cambridge, Cambridge, UK

## Abstract

**Background:**

Development of high-throughput methods for measuring DNA interactions of transcription factors together with computational advances in short motif inference algorithms is expanding our understanding of transcription factor binding site motifs. The consequential growth of sequence motif data sets makes it important to systematically group and categorise regulatory motifs. It has been shown that there are familial tendencies in DNA sequence motifs that are predictive of the family of factors that binds them. Further development of methods that detect and describe familial motif trends has the potential to help in measuring the similarity of novel computational motif predictions to previously known data and sensitively detecting regulatory motifs similar to previously known ones from novel sequence.

**Results:**

We propose a probabilistic model for position weight matrix (PWM) sequence motif families. The model, which we call the 'metamotif' describes recurring familial patterns in a set of motifs. The metamotif framework models variation within a family of sequence motifs. It allows for simultaneous estimation of a series of independent metamotifs from input position weight matrix (PWM) motif data and does not assume that all input motif columns contribute to a familial pattern. We describe an algorithm for inferring metamotifs from weight matrix data. We then demonstrate the use of the model in two practical tasks: in the Bayesian NestedMICA model inference algorithm as a PWM prior to enhance motif inference sensitivity, and in a motif classification task where motifs are labelled according to their interacting DNA binding domain.

**Conclusions:**

We show that metamotifs can be used as PWM priors in the NestedMICA motif inference algorithm to dramatically increase the sensitivity to infer motifs. Metamotifs were also successfully applied to a motif classification problem where sequence motif features were used to predict the family of protein DNA binding domains that would interact with it. The metamotif based classifier is shown to compare favourably to previous related methods. The metamotif has great potential for further use in machine learning tasks related to especially *de novo *computational sequence motif inference. The metamotif methods presented have been incorporated into the NestedMICA suite.

## Background

A central goal in modelling genome regulation is the identification of transcription factors (TFs) and their target DNA binding sites, expressed as short nucleotide sequence motif models. This goal is becoming tractable even for higher eukaryotic genomes due to the availability of annotated reference genomes for numerous organisms, large scale protein-DNA interaction and gene expression studies, and advances in regulatory binding site motif inference algorithms.

Computational work to infer short DNA motifs has resulted in publication of literally hundreds of algorithms for approaching the problem (reviewed in [1,2]). Perhaps most important computational advances in the field of DNA-motif inference are the introduction of scalable motif inference methods that can be used for *de novo *regulatory motif inference on a genome scale, with annotated reference genome sequences as input [3-5], and the effective use of supporting evidence such as gene expression microarrays [6,7] or chromatin immunoprecipitation microarray (ChIP-chip) [8,9] or ChIP-seq data [10,11]. Several high throughput DNA-protein interaction assays have also been introduced during recent years. These include bacterial one-hybrid assays [12] and protein binding microarrays [13].

The most widely applicable model for short regulatory motifs is the position weight matrix (PWM), originally introduced by Stormo et al [14]. The PWM is a model for gapless position-specific probability distributions of nucleotides which assumes independence of nucleotide positions [15]. Departures of the position independence assumption have been reported in the form of variable length linkers, interdependencies between nucleotides at different binding site positions [16-18], and compensatory mutations that maintain the binding energy and function of binding sites [19]. More complex probabilistic motif models based on for instance Bayesian [20,21] and Markov networks [22] have been developed to fit these observations. With the exception of the newest DNA-protein interaction assays which provide deep coverage of DNA interactions of proteins [23], parameter estimation of motif models more complex than the PWMis however hard with often scarce biological data. The PWM therefore remains the model of choice for most large scale motif inference tools: it is intuitive to interpret as a sequence logo [24] and retains more of the information contained in binding site patterns than sequence word based models [25].

Methods have also been developed for comparing and clustering motifs. One application of clustering is to infer information about possible function, such as likely binding partners. This is especially valuable for motifs from *de novo *inference methods which generally do not offer a predicted function for the signals they report. One specific application of clustering methods is the labelling of motifs with the protein DNA binding domain most likely to bind them. This is based on the observation that the sequence specificity of structurally related binding proteins is similar. Familial binding profiles (FBP) offer one early solution [26]. FBPs are essentially average motifs derived from multiple alignments of motifs optimised to minimise the sum of squared deviations (SSD) between aligned motif columns amongst a familial multiple alignment of PWMs. FBPs for common transcription factor families are available through the JASPAR motif database [27].

Motif clustering methods however suffer from the absence of a natural distance metric between motifs; different metrics are thought to provide complementary information of motif relatedness. A *χ*^2^- based distance metric was found an effective measure by [28]. A metric based on Pearson correlations of motif columns was also described in the same publication. Clustering based on various other distance metrics was suggested and systematically evaluated by [29]. A sum of squared deviations based metric was found to be the best single metric in this systematic comparison of a number of distance methods. The asymptotic covariance between hits of two motifs in a sequence has also been applied as a distance measure [30]. The most recent motif distance metric and clustering methods are probabilistic and draw special attention to the uncertainty in motif comparison and the importance of high-information columns in measuring distances of sequence motifs: a Bayesian probability distance metric between motif columns [31] and a fuzzy integral based metric [32]. In this work we also explore a probabilistic solution for the problem of comparing motifs.

Supervised learning strategies have also been applied to classify motifs and infer motifs similar to previously known motifs from novel sequences. Neural networks have been applied for classification of binding sites for the purposes of motif inference by [33]. Other notable methods include a Sparse Multinomial Regression (SMLR) based binding site sequence classification described in [34], and an application of this method for motif inference; The motif inference program PRIORITY assigns an SMLR- derived prior probability for each sequence position for its potential to fit a motif of a given transcription factor family [35].

We present here a probabilistic model for describing motif families and measuring relatedness of sequence motifs. We show sequence motif related machine learning uses for the model. At its simplest, a metamotif can be used to summarise gapless alignments of motifs of a given length, similarly as the FBP described above. As opposed to the FBP, we however do not model the recurring patterns found amongst a related set of motifs as a single motif alignment model but allow multiple components to describe a motif family.

We model a motif family as a series of motif components ("metamotifs") where different positions of a PWM motif can potentially map to different metamotifs. Not all positions are generated from a metamotif but some are treated as noise emitted by a background model. The metamotif includes a vector of column wise mean nucleotide weights, as well as variance for each column which is not modelled for example by the FBP. In this respect a metamotif is similar to the model used by MotifPrototyper [36]: both describe familiar prototypes of PWMs that are estimated probabilistically with a sequence of position specific probability distributions and can yield a Bayesian prior on the weight matrix columns (a 'structural prior' for the weight matrices in the terminology used by Xing *et al*.). In contrast to MotifPrototyper, the metamotif inference algorithm we developed can account for intra-motif structure such as repeating or palindromic segments by treating motifs as a series of several metamotifs and background positions.

## Results and Discussion

**Figure 1:**
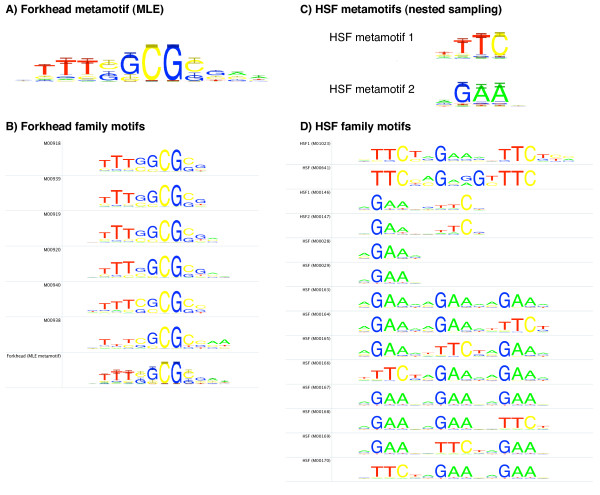
**example metamotifs for A) forkhead and B) HSF motif families**. A) The MLE metamotif estimated for a subset of forkhead motifs (B) in the TRANSFAC 12.2 [37] regulatory motif database. C) Two HSF metamotifs estimated using the metamotif nested sampling algorithm from a subset of HSF motifs (D) in the TRANSFAC regulatory motif database.

A metamotif is a generative model for PWM motif columns that can be used to represent a gapless alignment of position weight matrices. For each PWM position *i *(multinomial column) there exists a Dirichlet distribution in the metamotif column at position *i*. A metamotif is therefore a parameter configuration for a product Dirichlet distribution where position *i *of the motif alignment model corresponds to parameters *α*_*i*_. More intuitively, a metamotif of length *k *is a probability distribution over PWM motifs of length *k *(Figure 1A, C). Sampling motifs from the metamotif is also possible (samples drawn from a forkhead metamotif are shown in Additional File 1 Figure S1). This is analogous to computing a probability score for a sequence *k*-mer to measure the probability of the *k*-mer having been generated by a PWM, and drawing *k*-mer samples from a PWM.

Below we first describe the metamotif in detail. We then expand the use of the model beyond simply constructing metamotifs as global gapless alignment models of motif sets. This expansion is made possible by a Markov Chain Monte Carlo (MCMC) metamotif inference algorithm that simultaneously estimates multiple weakly represented metamotifs from a potentially large set of motifs. Illustrative examples of metamotifs are shown in Figure 1 alongside the input motifs for which the metamotifs were estimated.

### The metamotif

A metamotif *α *is a matrix of *L *columns, each defining a Dirichlet distribution over ℝ^*K *^where *K *is the size of the alphabet (Equation 1).(1)

A motif **X **= (**x**_1_, **x**_2_,...,**x**_*n*_) is a set of column vectors over the same alphabet. The probability of observing the column **x**_*i *_from the metamotif *α *is given by the density of Dirichlet distribution with parameters *α*_*i *_at weights **x**_*i*_(Equation 2). The normalising constant *B*(*α*) is the multinomial beta function, expressed in Equation 3 via the Gamma function.(2)(3)

The log probability of observing a motif of length *L *is then given by Equation 4.(4)

To motivate the use of the metamotif we note that the metamotif column *α*_*i *_can be understood as a combination of the mean nucleotide weights [*x*_*mk*_] and precision  (Equation 5).(5)

We have developed a visualization for the metamotif akin to the sequence logo. Our visualization presents both the mean weights [**X**|*α*] and precision *α*_0 _aspects of the metamotif. A sequence logo is drawn for PWM with nucleotide weights [**X**|*α*]. Error bars are shown to highlight 95% confidence intervals of nucleotide weights of the Dirichlet density at *α*_*i *_for each symbol *i, j *(Figure 1).

### Metamotif inference by nested sampling

The metamotif can be seen as a way to summarise a gapless alignment of motifs of a certain length, to yield a probability distribution of motifs. However, our goal in designing the metamotif framework was to describe recurring patterns seen in sequence motif data deposited in public motif databases such as TRANSFAC [37], JASPAR [38] or UniPROBE [39]. Many sequence motif families cannot be described accurately by global gapless multiple alignments of motifs at a fixed length. Motifs can for example consist of shorter repetitive signals, such as in the case of the heat-shock factor (HSF) motifs (Figure 1D), or the basic Helix-Loop-Helix (bHLH) motif family that are completely or partially palindromic due to their dimeric binding mode [40]. Inspection of the HSF motif set shows that a global alignment of its columns does not describe the regularly spaced fivebase repeat that is observed as part of the motifs in opposing orientations (aGAAn/nTTCt) [41]. Furthermore, even non-repetitive and non-palindromic motifs present challenges for gapless multiple alignments: the span of informative columns contributing to familial patterns in publicly available PWM data is often unclear because of different signal-to-noise ratios and varying information content criteria used for calling motif ends.

We wanted to develop an inference algorithm that allows simultaneous detection of *n *short metamotif signals from a set of motif data, allowing for varying length for different metamotifs, and optionally free orientation (signal present on either strand). The metamotif count *n *is a fixed, user settable parameter to the algorithm. For metamotif inference problems where *n *is expected to be large, the choice for the parameter should be informed by prior information of the motif set under study, for example clustering of the motifs to estimate a rough number of recurring motif segments. Each metamotif has *a priori *an unknown length between *l*_*min *_and *l*_*max*_columns, and is expected to contain one or more matches in a fraction *f *of motifs. Motifs in the framework are thought to be generated by recurring metamotif patterns, each of which is potentially shorter than any of the motifs, and background positions that model "uninteresting" sections of the motifs (positions not emitted by any of the metamotifs). The background model in the framework is the maximum likelihood (MLE) Dirichlet distribution estimated from all the motif columns in the input data. It is computed with the optimisation procedure described in [42].

Our metamotif inference algorithm is a variant of the NestedMICA nested sampling algorithm described in detail in [43]. Nested sampling, originally introduced by [44], is a generic Bayesian MCMC sampling strategy that allows drawing samples from a posterior distribution and directly estimating the evidence (marginal likelihood) of the model. In brief, nested sampling operates on an ensemble of states ranked by their likelihood. The initial state of the algorithm is drawn by sampling uniformly from the prior distribution. At each iteration, the state with the lowest likelihood is removed from the ensemble and replaced with a new state whose likelihood is bounded to be higher than that of the removed state. New states are created either by drawing samples from the prior distribution of states, or by decorrelating other randomly chosen states in the ensemble.

The metamotif inference algorithm allows estimating *n *metamotifs for a set of *p *motifs, with a variable metamotif length between *l*_*min *_and *l*_*max *_columns, and an expected fraction *f *of motifs containing any one of the *n *metamotifs. This is analogous to the NestedMICA motif inference algorithm that estimates multiple motifs with varying length from an expected fraction of nucleotide or protein sequence data. The posterior distribution being sampled is over the sets of *m *metamotifs and so-called mixture matrices, given the motif data and a background model for the motifs. The mixture (or occupancy) matrix describes the pairing between metamotifs and motifs. The term mixing matrix is a reference to the algorithm treating pattern recognition as an independent component analysis problem: a likelihood function is written for the observations (the motif set) and the motif set is assumed to be generated as a mixture of independent metamotif contributions and noise represented by the background model. Each element **Q**_*i*, *j *_in the *n *× *p *mixing matrix **Q **is a binary indicator of the metamotif *j *being present one or more times in the motif *i*. If the metamotif is present, **Q**_*i*, *j *_= 1, otherwise **Q**_*i*, *j *_= 0. The likelihood of the motif set given the metamotif set is simply the product of likelihoods of each individual motif given the metamotif set and the mixture matrix.

Likelihood of a motif given a set of metamotifs is calculated assuming the motif is emitted by a Hidden Markov Model (HMM) we call the multipleuncounted motif-metamotif HMM mixture model, or MUMM (MUMM with metamotif count 2 is given in Additional file 1 Figure S2). This formulation allows for each motif to contain multiple metamotifs simultaneously, without the need to iteratively repeat sampling after masking previously inferred signals. Computing the likelihood of a motif given metamotifs under the MUMM model involves completing one-dimensional dynamic programming from the beginning of the motif to column *c*, closely in the same form as the protein or nucleotide sequence likelihood function described in [43] (Equation 6).(6)

*L*_*c *_represents the likelihood of all metamotif and background column arrangements (paths) in the input motif up to the column *c*. *M *is the set of metamotifs that have a mixing coefficient of 1 for the motif under consideration (i.e. metamotifs marked to be present in the motif in the mixing matrix **Q**), and |*M*| is the number of metamotifs that have a mixing coefficient 1. The length of the metamotif *α *is represented by *l*_*α*_. *B*_*c *_is the probability that the motif column at position *c *was emitted by the background. For the motif **X **of length *l*_*X *_the transition probability *t *to a metamotif is defined as *t *= 1/*l*_**X**_, i.e. one metamotif is expected per motif, and any motif position is equally likely to contain a transition.  is the probability that the motif segment from *i *to *j *was emitted by a metamotif *m*, and it is given by the metamotif density function (Equation 4). A metamotif can optionally be allowed to be present on either strand to improve our ability to detect repeating (e.g. palindromic) features. Alternating orientation of metamotifs are achieved simply by summing the probability contributions  of the metamotif *α *and its reverse complement at all possible offsets.

Accounting for incomplete metamotif matches in a motif is an important consideration. This is because we wish to analyse data from different experimental and computational sources where motif start or end positions have not been chosen consistently, for instance with an information content criterion. Incomplete hits are accounted for by adding *l*_*max *_additional "un-informative" columns in the input motifs in both the 5' and the 3' motif ends. The un-informative columns are multinomial distributions that match the mean nucleotide weights of the background model Dirichlet distribution. This effectively allows all possible offsets of the metamotif that overlap the motif with at least one column, whilst associating more uncertainty to those columns supported by only a subset of the motif data.

Performance of the metamotif inference algorithm was tested using synthetic motif sets where samples from known metamotifs were inserted, or "spiked". The aim was to measure the relative frequency of metamotifs at which the expected metamotifs could be recovered by the algorithm from synthetic motif data containing metamotif instances. The evaluations were done in two stages. The ability of the algorithm to infer a single metamotif presented to it was tested first. After that, several metamotifs were presented to the algorithm to assess the ability of the algorithm to infer multiple metamotifs simultaneously.

To prepare the synthetic motif sets, metamotifs were first generated of examples of three structurally diverse TRANSFAC 12.2 PWM families: six forkhead motifs (class 3.3 in TRANSFAC classification), six GATA-like Cys_4 _zinc finger motifs (class 2.1) and five MADS box motifs (class 4.4) were used (source motifs shown in Additional file 1 Figure S3). This was done by aligning the input each of the three motif sets with a greedy gapless sequence motif multiple alignment method similar to the one utilised in STAMP motif toolkit [45]. A metamotif was then estimated from the motif multiple alignments with the MLE method from [42]: MLE Dirichlet distribution was computed for motif alignment columns (example seen in Figure 1A), with each motif column in the alignment mapping to a MLE Dirichlet distribution in the resulting metamotif.

Motifs (PWMs) from each of the three familial metamotifs were sampled in relative frequencies of 0%, 10%, 20%,...,100%, into synthetic input motif sets (separate input motif set per motif family). Each synthetic motif set contained 60 motifs, each 20 nucleotide columns long, with a maximum of one metamotif instance allowed per input motif. The synthetic motif columns in the input motif sets are samples from a Dirichlet distribution with parameters *α *= {0.5, 0.5, 0.5, 0.5}. The metamotif sample PWMs were inserted at random positions within the 20 nucleotide long synthetic motifs (example GATA motif set given in Additional file 1 Figure S4A,B,C). The metamotif inference algorithm was then run on the motif set to infer a single metamotif between length ranges 4 and 14, allowing for the signal to be present in either orientation (-numMetamotifs 1 -revComp -minLength 4 -maxLength 14).

**Figure 2:**
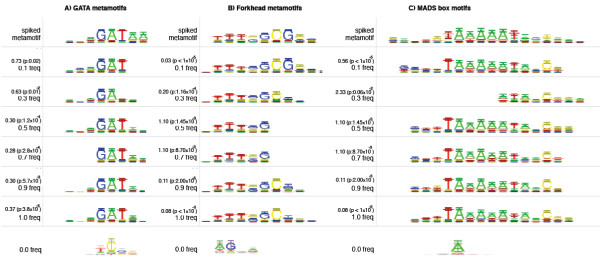
**Metamotif estimation from simulated motif data**. Metamotifs estimated with the metamotif nested sampler algorithm with varying relative frequency of metamotif samples. The top row in each metamotif alignment contains the "correct" metamotif that was sampled to the input weight matrix data in six different relative frequencies: 0.0, 0.1, 0.3, 0.5, 0.7, 0.9, 1.0. Frequency 0.0 which is shown in the bottom of the graph refers to a control experiment where all columns of the motif set are samples from the background (a Dirichlet distribution with parameters *α *= {0.5, 0.5, 0.5, 0.5}). A Cartesian-like distance between the sampled metamotif column mean nucleotide weights of the shown metamotif and the spiked metamotif mean nucleotide weights is presented above the relative frequency. An empirical *p*-value as described by [3] is also shown for the Cartesian distances (100,000 shuffles made for each motif).

Metamotif inference performance was measured qualitatively with visual inspection comparing the inferred metamotifs to the known spiked metamotifs, and quantitively measuring the Cartesian distance between the metamotif mean nucleotide weights. The visual comparison, Cartesian distances and empirical *p*-values for observed metamotif-metamotif distances are presented in Figure 2. The evaluation shows that metamotifs can be inferred from motif sets that contain them with relative frequencies of even 10%. At a relative frequency of 40% and above all three recovered metamotifs are found to be relatively similar to the respective source metamotif (Figure 2), although missing columns from metamotif ends are seen up to the relative frequency of 70% in the case of the forkhead and GATA motif families.

The ability to predict multiple metamotifs was demonstrated in a second evaluation experiment where instances of all the three motif families were inserted into synthetic motif sets and the algorithm was required to infer three metamotifs. It was shown that the algorithm was able to infer multiple metamotif models concurrently at a relative frequency as low as 20% (Additional file 1 Figure S5).

**Figure 3:**
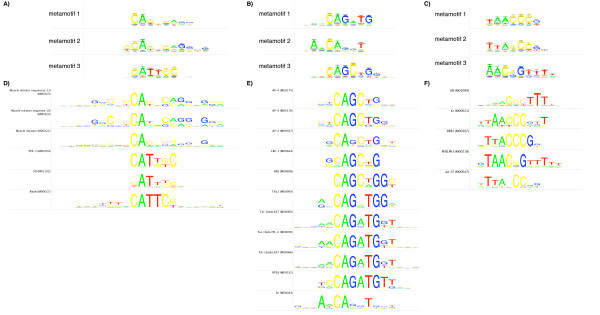
**Metamotif inference from clustered TRANSFAC motifs**. Examples of metamotifs inferred with the metamotif nested sampler algorithm from clustered motifs deposited in the TRANSFAC database [37]. Three metamotif sets (A,B,C) were estimated from input motif sets (D, E and F, respectively). A) Two largely similar patterns (metamotifs 1 and 2) are inferred that correspond to the muscle initiator like motifs, with one distinct pattern (metamotif 3) corresponding to the CATTCC-like motifs (members of the TEA family) also found from the cluster. B) shows that the metamotif nested sampler distinguishes a CAGATG-like (metamotif 1) and CAGCTG-like pattern (metamotif 2) from the motif cluster 3E, and separates an Sn (M00044)-like outlier from the two patterns as metamotif 3. C) Three metamotif patterns inferred from the motif set separate the TAACCCG-like (metamotif 1), TTACCCG (metamotif 2) and TAACCGTTT (metamotif 3) like patterns seen in the motif cluster. Notably metamotif3 contains a shorter CCG-like core instead of the CCCG found in the two other metamotifs.

We demonstrated use of the metamotif nested sampling algorithm in inferring familial metamotifs from known experimentally determined regulatory motifs from the TRANSFAC database [37]. Motifs retrieved from TRANSFAC were first divided to clusters with the SSD distance metric described in [3] with cutoff 6.0. Three metamotifs were then inferred from each of the resulting clusters. Examples of metamotifs inferred are shown in Figure 3. The metamotif nested sampler algorithm was found capable of detecting several recurring patterns from the motif clusters that are clear upon visual inspection of the motifs, in addition to finding overliers from the motif sets (Figure 3B).

Our results show that the metamotif nested sampler algorithm, the first metamotif inference algorithm to be described, can discover detailed structure from motif sets. We show the algorithm is capable of correctly inferring multiple familial metamotif patterns. This makes the algorithm applicable for example for finding redundant motif patterns from large scale *de novo *inferred motif predictions from different algorithms, or for inferring a complete set of familial metamotifs from a set of motifs.

### Metamotifs as a position weight matrix prior

*De novo *gene regulatory motif inference algorithms commonly suffer from lack of sensitivity when applied to large collections of long eukaryotic promoter sequences, rendering it impossible to describe complete sets of regulatory motifs with sequence alone. We therefore wanted to see if prior biological knowledge in the form of familial metamotifs could be used to improve the sensitivity of a motif inference algorithm. This was motivated by the work by [35] which showed familial tendencies in the motifs of sequence specific transcription factors can improve motif inference sensitivity. We therefore wanted to test if a probabilistic model of the observed spectrum of DNA specificity of known transcription factor motif families expressed as a metamotif could be used to improve the sensitivity of motif inference from novel sequence.

We extended the NestedMICA motif inference algorithm to accept a series of metamotifs as a position specific prior probability function for motifs. The NestedMICA algorithm was chosen for the purpose as it is known to perform well in large scale motif inference tasks [3,46], and because the previously published version of the algorithm applies an uninformative Dirichlet prior probability function to motif columns (all allowed nucleotide weight combinations equally probable *a priori*), which made it straightforward to change the algorithm to take use of column-specific informative Dirichlet distributions.

The prior probability of motif **X **given a metamotif *α *is taken as the sum of metamotif densities of *α *with all continuous motif segments contained in **X **that have the same length *l *as the metamotif (log of the density is given by Equation 4). A segment of motif **X **refers to a motif formed from columns of the motif starting from column *i *and ending at position *i *+ *l *- 1. The prior probability of a motif given series of metamotifs is simply the sum of prior density contributions of each of the metamotifs.

To test the performance of the metamotif prior, we conducted simulation experiments following the same principle as described for the NestedMICA [46] and the BayesMD [47] algorithms. Human intronic nucleotide sequence fragments randomly chosen from the *Homo sapiens *Ensembl database release 50 [48]) were 'spiked' with five different types of motifs. The motifs used were those of ZAP1, HIF1, TBX5, TAL1 and NF-*κ*B transcription factors. These motifs were selected because they showed little similarity with each other when aligned, and because this set contains examples of differing motif length and information content. All sequence sets used contained 200 sequences, and the total length of the sequence was varied between 100, 200,...,2000 nucleotides. The nucleotide *k*-mers sampled from each of the five PWMs in the evaluation were inserted at a constant relative frequency of 20% of the sequences, with a maximum of one motif present per sequence. In other words, motif density was varied by inserting the motif instances to sequences of different lengths. Motifs of only one kind were present in each synthetic sequence set.

Motif inference with three types of prior functions were tested with the sequences: firstly a single familial metamotif contributing to the prior function was used. Secondly we tested a prior function with all of the five unrelated metamotifs contributing to the prior, with instances of only one motif family being actually present represented in the sequences. Thirdly, we used an uninformative PWM prior similar to the previously published NestedMICA version 0.8. In each of the motif inference runs, the longest sequence length at which the algorithm infers the correct motif of interest is reported as a measure of sensitivity (*p *< 0.05), with motif comparison *p*values computed as described in [3]. In all cases, five motifs were inferred from the sequences (as recurring sequence motifs tend to be found from intronic sequences, we cannot assume that the spiked motif would be the only signal present). The sequence background model used in all evaluations of the algorithm was a 4-class 1^*st *^order trained from the 2000 nt long intronic sequences with nmmakebg.

**Figure 4:**
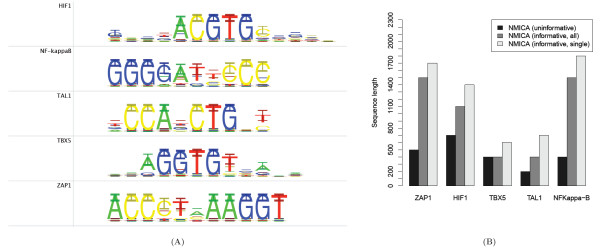
**The effect of metamotif position weight matrix prior on motif discovery with Nested MICA**. A) Metamotifs used as the motif priors. Error bars represent 95% confidence intervals. B) Informative weight matrix prior improves NMICA's sensitivity to resolve motifs present in human intronic sequence in low frequency (0.2 frequency). The bars represent the sequence length at which a motif closely similar to the input motif was successfully recovered (*p *< 0.05, empirical *p*-value defined in [3]).

The source motifs (ZAP1, HIF1, TBX5, TAL1, NF-*κ*B) were transformed to metamotifs to be used in the metamotif prior function by applying a pseudocount of 0.1 to the motif column weights, and interpreting the resulting motif nucleotide weights as mean nucleotide weights in Dirichlet distributions with precision set at 4.0 (metamotifs used in the experiment shown in Figure 4A). The metamotif priors used in the prior function evaluation were constructed from known PWMs with a set precision and pseudocounts to test the most common hypothesis testing use of a motif prior function: user is aware of a set of potentially relevant motifs or consensus strings present in a sequence set and wants to inform the algorithm of them to increase sensitivity.

The informative motif prior function was shown to dramatically improve the capacity of the Nested-MICA algorithm to resolve weakly represented sequence motifs presented to it in longer nucleotide sequences, especially those with larger number of columns and higher information content (Figure 4B). Both the baseline motif inference sensitivity and the effect of the informative weight matrix prior was seen to vary based on the length (likely due to the information content) of the motifs, ranging from as high as fourfold difference in the motif recovery length for TAL1 and NFKappa-*β*, to only a 1/3 improvement from 400 bp to 600 bp sequence between the uninformative and the 'single' informative metamotif prior for the TBX5 motif. Sensitivity to infer all but the TBX5 motif showed improvement with the combinatorial metamotif prior over the uninformative prior, with the effect less pronounced than when the prior function only contained a contribution from the correct metamotif. These results suggest that the metamotif prior is performing as hoped, and multiple metamotifs can be combined to a 'combination metamotif prior function' to detect motifs where information is available of candidate motifs.

We wanted to ensure that the metamotif prior did not have the propensity to bias motif inference to an incorrect solution, i.e. that it does not encourage the inference of a motif not supported by the sequence data. We tested this by spiking intronic sequence with the NF-*κ*B motif, and using the ZAP1-like metamotif in the prior function. No motifs similar to ZAP1 (whose instances were not present in the sequences) were recovered from the spiked intronic sequence between lengths 100 and 2000 (comparison with distances and *p*-values shown in Additional file 1 Figure S6), indicating that the metamotif prior function does not have an adverse effect on inference specificity. A number of other combinations of spiked motifs and inaccurate informative metamotif prior functions were also tested, with no observed tendency for the algorithm to infer a motif that not supported by the sequence data (data not shown).

The metamotif prior extension to the Nested-MICA algorithm is designed to function with any number of metamotif models, input PWMs or IUPAC consensus sequences. PWMs are treated as metamotif priors by interpreting its columns *i *as the [**X**|*α*] of a metamotif and applying a constant precision *α*_0 _to all columns of the metamotif. IUPAC consensus sequences are first transformed to PWMs by applying pseudocounts and then transformed similarly as PWMs. Metamotifs inferred with our framework could also be applied to other Bayesian motif inference algorithms which model a prior distribution over motif columns. Metamotifs could therefore be useful in building large or even complete regulatory binding site motif libraries for novel genomes.

### Predicting regulatory motif type with metamotifs

The metamotif is shown above to improve motif inference sensitivity dramatically when used as a Bayesian PWM prior. We however also wanted to test if metamotifs could be used to form functional predictions for novel motifs based on a database of previously known motifs. We developed a motif classification tool for the purpose, termed metamatti, for **metam**otif based **a**utomated **t**ranscription factor **t**ype **i**nference.

**Figure 5:**
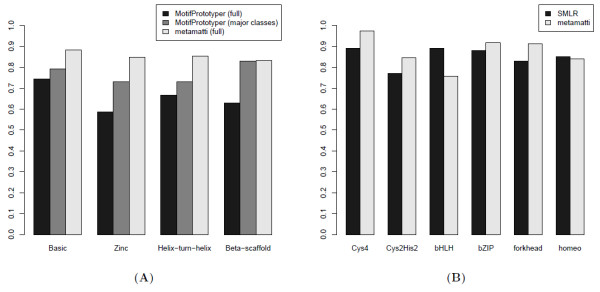
**Comparison of metamatti with MotifPrototyper and SMLR motif classification**. Accuracy comparison between A) TF domain superfamily classification with metamatti and MotifPrototyper (10-fold crossvalidation) and B) TF domain family classification with metamatti and SMLR (k-fold crossvalidation). The 'major classes' in (A) refers to MotifPrototyper's reported performance for all motif families which include at least ten motif instances in TRANSFAC [37] that belong to the four superfamilies (basic, zinc, helix-turn-helix and *β*-scaffold), and 'full' refers to a classification of all motifs in the four superfamilies. C) The expected metamatti homeodomain motif family classification accuracy is contrasted with the accuracy achieved with *M. musculus *protein binding microarray derived PWMs [51] and *D. melanogaster *bacterial one-hybrid [52] homeodomain motif datasets. Expected accuracy refers to the out-of-bag accuracy estimate of the metamatti motif classifier.

The principle of our motif classifier is to compute the density function (Equation 4) of a large dictionary of familial metamotifs along the length of training set motifs, effectively "scanning" weight matrices with metamotifs. The optimal (maximum) and average metamotif densities of each metamotif with the motif are then extracted as features in a random forest classifier that tries to infer the TRANSFAC superfamily (Figure 5A) or TRANSFAC family (Figure 5B, C) of the motifs. Random forest classification was chosen as the machine learning framework because it generalises naturally to multi-class problems and provides unbiased classification error estimates as part of model training [49]. The framework also automatically controls the sparsity of the feature set that contributes to the classification. The full list of feature types used in the metamatti classifier are shown in Table 1.

**Table 1:** Features calculated for motifs in the metamatti classifier

Feature	Description
Maximum metamotif hit scores with all of the familial metamotifs	Motifs were scanned with all input metamotifs and the optimal score was chosen. Both familially discovered and models discovered.

Per-column average entropy	Average Shannon entropy of columns.

MLE Dirichlet parameters	A maximum likelihood Dirichlet distribution is estimated as described in [42] and the parameters of this distribution are used as features (*α*_*A*_,*α*_*G*_,*α*_*C*_,*α*_*T*_).

Symmetric Dirichlet background parameters	A symmetric Dirichlet distribution is estimated as described above.

All motif families with at least 10 representatives were retrieved from the TRANSFAC 12.2 database [37], totalling 623 motifs of 13 domain families. For the motif domain superfamily classifier comparison made with MotifPrototyper [36] (Figure 5A), the set of motifs was reduced further to include only motifs annotated in TRANSFAC with the four superfamilies classified in [36]. Similarly, for the motif family classification comparison with SMLR [34] (Figure 5B), only motifs of the same six major classes classified in [34] were included in our training set.

Most features in the classifier are metamotif probability density scores of the training set motifs with metamotifs trained from motifs of each of the motif families. To compute the density features, we chose to first divide the motifs of each family into smaller clusters by complete linkage hierarchical clustering them with the SSD metric described in [3] and cutting the clusters at a lenient clustering cutoff of 6.0, resulting in 65 motif clusters. Three metamotifs were trained from each motif cluster with nmmetainfer, resulting in 195 metamotifs to be used in the motif classifier (examples seen in Figure 3). Metamotif length was constrained between 6 and 15 columns, and the expected usage fraction was set at 0.5.

Motif classification performance of metamatti was compared to two methods with a related goal: MotifPrototyper [36] which classifies motifs into four TRANSFAC superfamilies (zinc coordinated, helixturn-helix, *β*-scaffold,basic), and SMLR which classifies motifs into six major classes of TF domains (Cys_2_His_2 _and Cys4 zinc fingers, homeodomains, forkhead domains, basic helix-loop-helices and basic zipper domains) [34].

Classification accuracy comparison shows that metamatti outperforms MotifPrototyper [36] (Figure 5A) across all motif superfamilies. There are several possible reasons for the difference in performance. Firstly, the metamotif inference algorithm is not constrained to a fixed motif family model column count, unlike the algorithm utilised in MotifPrototyper. Secondly, training several metamotifs per motif family, metamatti also accounts for the fact that not all columns in motif families can be accurately expressed as a single column wise probability distribution. Instead, recurring patterns in a motif set can be generated by multiple potentially shorter familial metamotif components in our model. Thirdly, the metamotif estimation algorithm treats some motif columns as noise with a column background model, improving our capacity to find the recurring patterns from sequence motif sets and reducing over-fitting of familial models due to reporting weak or nonexistent recurring trends.

Motif family level classification was conducted with the same subset of TRANSFAC 12.2 PWMs that were classified with SMLR by Narlikar and Hartemink [34]. The overall classification accuracy comparison shows that metamatti has a marginally improved performance at 89.5% classification accuracy over the 87% reported for SMLR. However, the class-by-class accuracy figures (Figure 5B) and the confusion matrix of the 6-way TRANSFAC motif family classifier (Table 2) make it evident that the higher classification accuracy comes at the cost of a 14% drop in the classification accuracy of the bHLH family (89% accuracy with SMLR, 75% with metamatti). The E-box motif CAGGTG appears to be the most common type misclassified in the erroneous bHLH motif cases. Inspection of motif family assignments in the TRANSFAC database shows that closely similar motifs with the CAGGTG consensus have been annotated with all of bHLH and C2H2 zinc finger families. In fact it has been proposed that certain bHLH and Snail-like C2H2 like factors are thought to bind with closely similar specificities to compete for the same binding site positions [50], highlighting a general limitation of a sequence PWM feature based motif family classification methods.

**Table 2:** Confusion matrix of the 6-way TRANSFAC motif classification

	Cys4	C2H2	bHLH	bZIP	Forkhead	Homeodomain	Class error
Cys4	39	0	0	0	0	1	0.025

C2H2	0	38	3	0	1	3	0.156

bHLH	0	2	22	5	0	0	0.24

bZIP	0	3	0	78	0	4	0.08

Forkhead	0	0	0	0	31	2	0.09

Homeodomain	2	1	1	3	0	37	0.16

**Totals**	41	43	26	86	32	47	

Clustering of the motifs and training metamotifs from motif clusters was motivated by the requirement to choose a value for the metamotif count parameter of the metamotif inference algorithm, and to limit the metamotif search space. Inspection of clusters at cut off 6.0 showed no clusters with more than three strongly distinct recurring patterns. Although for many motif clusters there were clearly less than three distinct recurring metamotif patterns present at the clustering cutoff of 6.0, the metamotif inference algorithm was found to treat these cases by either inferring closely similar duplicate metamotifs (such as metamotifs 1 and 2 in Figure 3A) or short metamotifs with mean nucleotide weights with low information content, or occasionally splitting the metamotif segments in several independent parts. This suggested to us that that together with a sparse machine learning strategy such as a random forests, it would be advantageous to choose a high metamotif count that described the input motif set in as much detail as possible, with the price of some potentially redundant features in the feature set (densities for duplicate or low information metamotifs). We validated this assumption by retraining the classifier with two metamotifs per cluster (a total of 130 metamotifs). The classifier trained with two metamotifs per family resulted in a mild decrease in the classification accuracy (88.4%, as opposed to 89.5% with three metamotifs per cluster), suggesting that the additional metamotifs were indeed informative.

We also wanted to assess the significance of the metamotif density score in the metamatti classifier by comparing it to a more naive classifier where we replace the metamotif average and maximum scores with average and maximum SSD distances computed between the training set motifs and 'average motifs' of each of the motif family, instead of scoring training set motifs with familial metamotif density average and maximum scores. The average motifs we used in the more naive classifier were the mean PWMs of the metamotifs trained with nmmetainfer were used for classification by scoring the training set motifs with an SSD distance metric with each of the metamotifs. We found that the classifier accuracy achieved with the SSD metric was lower to the metamotif density based classifier by 1.4% (accuracy of 88.1%), suggesting that both the metamotif mean and the column wise precision values which contribute to the metamotif density scores are partially responsible for metamatti's high performance. Furthermore, we tested training a classifier with cluster average motifs instead of the metamotif segments, resulting in an accuracy figure of 86.5%, suggesting that not only is the metamotif density a suitable score, but that the motif segments identified by the metamotif inference algorithm provide a classifier that generalises better than simply using average motifs inferred by clustering and collapsing clustered motifs to an average representation.

### Independent validation of the TRANSFAC family prediction of motifs

Previous motif classification work has relied on separating training and testing data from a single public database using cross-validation [26,34,36], where most motifs originate from individual studies and therefore different experimental sources. Recent advances in protein-DNA interaction assaying have however resulted in several large high-throughput regulatory motif data sets becoming publicly available. We therefore wanted to assess the performance of metamatti with two homeodomain motif sets recovered from different species and via different experimental methods: *Mus musculus *protein binding microarray motifs [51] and *Drosophila melanogaster *bacterial one-hybrid motifs [52]. This evaluation also allowed us to compare the classification accuracy achieved with the two independent datasets to the accuracy expected based on the out of bag classification error estimate of the metamatti random forest classifier training for the homeodomain motif family.

We trained a metamatti classifier with all 13 motif families present in the TRANSFAC 12.2 with at least 10 examples per family (total of 663 motifs) for evaluating classification of the two homeodomain motif datasets. The accuracy reported for both *Drosophila melanogaster *and *Mus musculus *datasets was the fraction of motifs labelled as homeodomain instead of any of the 12 incorrect labels.

The classification accuracy rates for both homeodomain motif sets were shown to be high, and in good agreement with the out-of-bag accuracy estimate of 91.3% reported by the metamatti random classifier during classifier training: 92.1% and 91.7% of the homeodomain motifs in the Berger *et al*. (84 motifs) and Noyes *et al*. (177 motifs) sets were correctly classified, respectively. We studied the misclassified examples from the *Drosophila melanogaster *homeodomain datasets in more detail to see where the misclassified motifs lie in the homeodomain specificity group clustering presented in [52]. Interestingly, the misclassifications were shown to be atypical homeodomains which do not contain the canonical TAATTA core and fall amongst the smaller specificity groups described in [52]. The misclassified motifs included three TGIF-Exd-like motifs (Vis, Hth, Exd), two Iroquis-like (Ara, Mirr), one Six-like (Optix) and an outlier from the specificity group clustering (Additional file 1 Figure S7A). A similar trend of non-canonical homeodomains being primarily amongst the misclassified was also noted for the *Mus musculus *homeodomain motifs (Additional file 1 Figure S7B). This is most likely explained by atypical homeodomain motifs not being well covered well by the TRANSFAC 12.2 training set; No closely matching homeodomain motifs were observed in TRANSFAC 12.2 to many of the misclassified motifs.

We also wanted to test if a metamatti-like classifier could be trained to detect more detailed differences between motif groups than motif family or superfamily. We therefore labelled the *Drosophila melanogaster *homeodomain motifs with the homeodomain specificity groups suggested by the Noyes *et al*. (2008) and estimated a single metamotif with nmmetainfer from each of the 11 specificity groups. We then trained a metamatti classifier with these metamotifs similarly as described above for the motif family and superfamily classifiers. We observed the remarkably high accuracy of 84% (confusion matrix shown in Table 3), when all Noyes *et al*. (2008) homeodomain motifs with 3 or more examples per specificity group were included in the classification (10-way classification). The applicability of supervised machine learning strategies that aim to learn motif type labels more precise than the DNA binding domain family are however currently limited by the amount of available training data. For instance, the 84 motifs in the Noyes *et al*. (2008) dataset contain examples of 11 specificity groups which are very biased to the two largest groups (Antennapedia and Engrailed, with 25 and 15 examples, respectively), with several specificity groups containing as few as two to four examples (Ladybird, Iroquis, NK- 1, NK-2, TGIF-Exd, Bcd). This makes classifier error estimation imprecise especially for the weakly represented classes, and results in the major classes which have as much as eightfold as many examples present in the training dataset to have considerable weight in predictions over the smaller classes (such as to maximise overall classification accuracy). Methods like metamatti can however become increasingly relevant once more high-throughput TF DNA specificity data becomes available.

**Table 3:** Confusion matrix of the homeodomain motif specificity group classifier

	AbdB	Antp	Bar	Bed	Engrailed	Iroquis	NK-1	NK-2	TGIF-Exd	Class error
AbdB	5	0	0	0	0	0	0	0	0	0.00

Antp	0	15	0	0	2	0	0	0	0	0.12

Bar	0	0	5	0	1	0	0	0	0	0.17

Bcd	0	0	0	4	0	0	0	0	0	0.00

Engrailed	0	1	1	0	23	0	0	0	0	0.08

Iroquis	0	0	0	0	0	3	0	0	0	0.00

NK-1	0	0	0	0	3	0	2	0	0	0.60

NK-2	0	0	0	1	0	0	2	0	0	0.33

Six	0	0	0	1	0	0	2	1	1	0.67

TGIF-Exd	0	0	0	0	0	0	0	1	3	0.25

**Totals**	5	16	6	5	29	0	4	3		

Confusion matrix showing the metamatti predicted classifications of the Noyes *et al*. (2008) homeodomain motif data. All classes with at least three representative examples were included in the classification (i.e. the two examples of the Ladybird specificity group were removed).

## Conclusions

We present a novel motif family model, the metamotif. We show its use as an informative prior in a motif discovery algorithm, and describe a motif classification method based on metamotif density features. We find that the method compares favourably to previously published related methods. Its performance with two novel experimental TFBS motif datasets is also found consistent with expected error estimates. The metamotif inference and visualisation tools are made openly available as part of the NestedMICA motif inference suite, and the interactive motif inference analysis environment iMotifs [53]. We also introduce a visual representation for the metamotif akin the sequence logo, which presents the metamotif as an average motif with confidence intervals for symbol weights.

We envisage that the metamotif will have further machine learning related uses in addition to the Bayesian prior and motif family classification method we present. Large scale computational motif inference frameworks especially could benefit from metamotif driven semi-supervised methods to either effectively estimate complete motif sets from novel sequence sets, or on the contrary to discriminatively infer motifs that do not not closely match known sequence motifs. Further large-scale profiling of eukaryotic transcription factor DNA specificity could also dramatically extend the data available for training metamatti-like motif classifiers that infer motif type labels, to allow more fine grained classification than the binding domain family or superfamily classification described in this work.

## Methods

### Metamotif inference by nested sampling

The metamotif inference algorithm evolves metamotif parameters and the mixture matrix state with Monte Carlo sampling moves. Most of the proposal types alter the metamotif column *α *parameters. The metamotif proposals are selected randomly from amongst the following set of moves:

• a small perturbation is made to a randomly selected metamotif column nucleotide mean weight: perturbation is made according to a randomly chosen nucleotide *α *weight *α*_*i*_, nucleotide mean weights adjusted so they again sum to 1, and *α*_*i *_of the column adjusted accordingly, maintaining precision unchanged.

• a small perturbation is made to a randomly selected metamotif column precision *α*_0_: *α*_0 _is perturbed, and *α *adjusted such as to maintain the mean nucleotide weights unchanged with a new precision.

• a small perturbation is made to a randomly selected metamotif column nucleotide weight *α*_*i*_, thereby indirectly changing the precision.

• replacing a metamotif column with a new one, sampled from an uninformative simplex prior (nucleotide weights on the range [0.1, 40.0] are allowed).

• removing a column in one end of a metamotif while adding another one to the other end.

• adjusting motif length, by adding or removing a column from either end.

The two update operations that use an alternative parametrisation of *α *with precision and the mean nucleotide weights, i.e. updating the precision whilst maintaining mean weights unchanged, and altering the mean weights whilst maintaining the precision unchanged, proved beneficial for achieving convergence of the algorithm. When these moves were included, the algorithm consistently converged with smaller number of iterations than when only the more naive method of updating *α*_*i *_with random perturbations was included (data not shown). The prior function over the Dirichlet distribution parameters was an uninformative 'clipped' simplex prior: all values for the nucleotide weight parameters *α*_*i *_of the distribution are allowed on the range [0.1, 40.0] and equally likely. Parameter values above or below this range are clipped such as to avoid numerical instability.

Sampling moves are also done in the the space of mixture matrices by flipping states of randomly selected elements in the mixture matrix similarly as is described in [43] for the NestedMICA algorithm.

The metamotif nested sampler algorithm was developed in Java and is made available in the Nested- MICA suite as a command line tool nmmetainfer. Because the parameters of the metamotif inference algorithmare highly similar to the motif inference algorithm in NestedMICA, the command line interface of nmmetainfer was also designed to be analogous to the motif inference tool nminfer included in the suite.

### Metamotif visualization

The metamotif visualization was implemented to the iMotifs sequence motif visualization environment [53] with the Mac OS X Cocoa drawing API (Objective-C programming language). Metamotif model visualization was also implemented in a Java based cross-platform motif visualization tool mXplor (see 'Availability and requirements' for more detail).

### Metamotifs as a position weight matrix prior

Human intronic sequences used in the metamotif prior performance evaluation were retrieved from the Ensembl database using the Ensembl Ruby API (unpublished, available from http://github.com/jandot/ruby-ensembl-api). Regions overlapping with annotated repeats called with Repeat-Masker [54] and dust [55] were rejected.

The PWM prior was implemented as part of the NestedMICA motif inference tool nminfer. A metamotif PWM prior can be supplied to nminfer either as an XMS formatted metamotif file with the argument -priorMetamotifs or as a an IUPAC consensus string (-consensus) with a set precision and pseudocount applied to the mean nucleotide weights (-priorPrecision and -priorPseudocount). NestedMICA motif inference was done in each case with the expected usage fraction parameter at 0.2, allowing for weight matrix occurences on either strand, and constraining the minimum motif length to 6 and maximum length to 14 (-expectedUsage Fraction 0.2-revComp -minLength 6 -maxLength 14).

The XMS file format that is used for exchanging motif data by NestedMICA and iMotifs is also used for representing metamotifs (see NestedMICA manual for more information). A cross-platform programming interface libxms is also available for reading and writing XMS formatted files for several common programming languages (Perl, Ruby, R, Java, Objective-C) [53] and the metamotifs can therefore be readily taken use of by other motif inference tools.

### Predicting regulatory motif type with metamotifs

The metamatti motif type classifier training and cross-validation were implemented in the Ruby and R [56] programming languages. Random forest classification was done using the package randomForest[57]. Pseudocounts of 0.01 were added to all training set metamotifs, and the *mtry *parameter of the random forest classifier training was optimised by testing 0.1 × , 0.2 × ..., 2.0 ×  with intervals of 0.1, where *p *is the number of features in the classifier (the default value for *mtry *is ). The *ntree *parameter that controls the number of trees to grow was set at 5000.

## Availability and requirements

The metamotif inference and metamotif PWM prior enabled version of the cross-platform Java based NestedMICA suite is available on the Nested- MICA project website at http://www.sanger.ac.uk/Software/analysis/nmica. The algorithms used in the metamotif inference validation experiment are also made available in the NestedMICA suite. These include the motif alignment tool nmalign (outputs the motif alignment as a FBP, set of aligned motifs, or MLE metamotifs), as well as the motif simulation tool nmmetasim.

Metamotif visualisation is made available as part of the Mac OS X based motif viewer iMotifs [53]http://www.github.com/mz2/imotifs, and the cross-platform Java based motif viewer MotifXplorer http://github.com/mz2/mxplor

All the above software is distributed under the Lesser General Public Licence (GPL) version 2.0 or later.

## Authors' contributions

TH, TD and MP conceived this work. MP implemented all the computational work. All authors read and approved the final manuscript.

## Supplementary Material

Additional file 1 **Figure S1 - a forkhead metamotif and individual forkhead-like motifs sampled from the metamotif**. A forkhead-likemetamotif (inferred from an alignment of motifs) is shown alongside selection of motif samples drawn from it. The wider error bars (representing 95% confidence intervals of nucleotide weights) of the thymine-rich 5' end of the metamotif is found consistent with the variation in the motif column heights. **Figure S2 - The motif-metamotif HMM**. The multiple-uncounted motif-metamotif HMM model (MUMM). Numbered steps model the columns of the metamotif signals of interest and the background states are responsible for the "uninteresting" positions. Motif columns are emitted from a selection of metamotifs of varying lengths, and background positions. The black dot is a silent state which does not model any part of the motif. **Figure S3 - the motifs used to create the target metamotifs for metamotif inference performance evaluation**. The presented motifs were aligned and the multiple aligned summarised as an MLE metamotif with the program *nmalign *(included in the NestedMICA suite). See the topmost metamotifs in Figure 2 for the resulting target metamotifs that were spiked into synthetic motif sets. **Figure S4 - example of a series of simulated motifs created for evaluating the metamotif inference algorithm**. An example of the simulated weight matrices generated for measuring the performance of the metamotif nested sampling algorithm. Samples from the GATA-like metamotif are present in 6 of the 60 sequence motifs (10% relative frequency). Similar sets were generated for frequencies between 0% and 100% for the three different structural classes of motifs studied in the simulation. **Figure S5 - simultaneous inference of multiple metamotifs**. The metamotifs predicted at relative frequency of 0.2 are shown alongside the source metamotifs. **Figure S6 - incorrect informative prior does not cause NestedMICA to report nonexistent motifs**. A metamotif prior was used in this experiment to inform of a ZAP1-like motif when in fact an NF-*κ*B motif is present in the sequences. We present the closest match between the ZAP1 motif (which is not present in the input sequences) and the inferred motifs (the closest match between the five motifs in the inferred sequence set). The distance measure and *p*-value shown are for comparison of the closest match with the ZAP1 motif, and they are described in more detail in [3]. **Figure S7 - comparison of misclassified homeodomain motifs to TRANSFAC 12.2 training motifs**. The motifs shown on red background are those homeodomain motifs misclassified by metamatti, with the incorrect class noted after the motif name. The motifs shown under each misclassified motifs are either the closest motif in TRANSFAC 12.2 to the misclassified motifs (in the three first cases). Click here for file
