# Supplementary material for: Metamotifs - a generative model for building families of nucleotide position weight matrices
Source: BMC Bioinformatics. 2010 Jun 25;11:348. doi: 10.1186/1471-2105-11-348 (PMC2906491; doi:10.1186/1471-2105-11-348)
Supplement: Additional file 1 — Figure S1 - a forkhead metamotif and individual forkhead-like motifs sampled from the metamotif. A forkhead-likemetamotif (inferred from an alignment of motifs) is shown alongside selection of motif samples drawn from it. The wider error bars (representing 95% confidence intervals of nucleotide weights) of the thymine-rich 5' end of the metamotif is found consistent with the variation in the motif column heights. Figure S2 - The motif-metamotif HMM. The multiple-uncounted motif-metamotif HMM model (MUMM). Numbered steps model the columns of the metamotif signals of interest and the background states are responsible for the "uninteresting" positions. Motif columns are emitted from a selection of metamotifs of varying lengths, and background positions. The black dot is a silent state which does not model any part of the motif. Figure S3 - the motifs used to create the target metamotifs for metamotif inference performance evaluation. The presented motifs were aligned and the multiple aligned summarised as an MLE metamotif with the program nmalign (included in the NestedMICA suite). See the topmost metamotifs in Figure 2 for the resulting target metamotifs that were spiked into synthetic motif sets. Figure S4 - example of a series of simulated motifs created for evaluating the metamotif inference algorithm. An example of the simulated weight matrices generated for measuring the performance of the metamotif nested sampling algorithm. Samples from the GATA-like metamotif are present in 6 of the 60 sequence motifs (10% relative frequency). Similar sets were generated for frequencies between 0% and 100% for the three different structural classes of motifs studied in the simulation. Figure S5 - simultaneous inference of multiple metamotifs. The metamotifs predicted at relative frequency of 0.2 are shown alongside the source metamotifs. Figure S6 - incorrect informative prior does not cause NestedMICA to report nonexistent motifs. A metamotif prior was used in this experiment [file 1471-2105-11-348-S1.PDF]

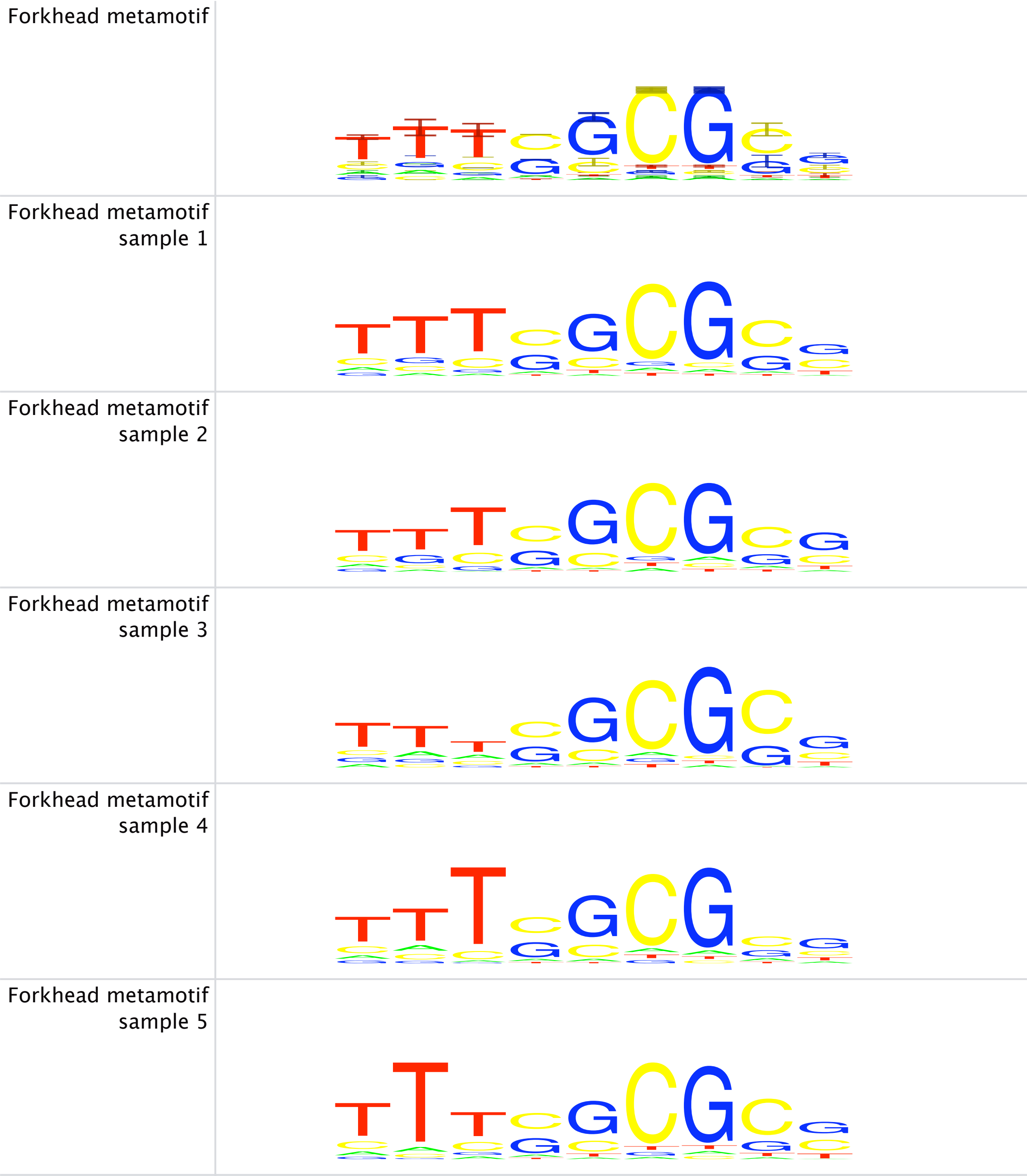
Forkhead metamotif
Forkhead metamotif sample 1
Forkhead metamotif sample 2
Forkhead metamotif sample 3
Forkhead metamotif sample 4
Forkhead metamotif sample 5

Figure S1.

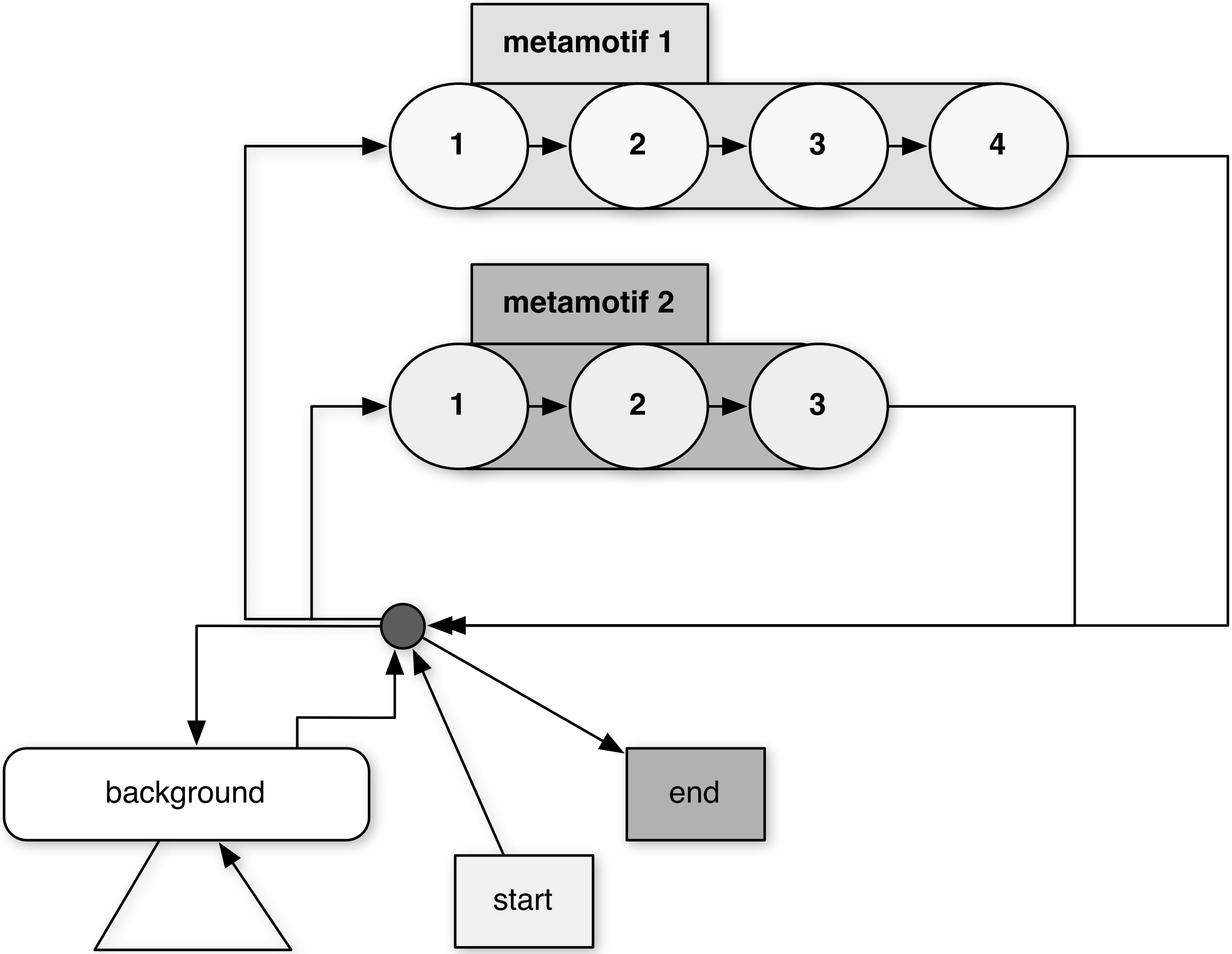

Figure S2.

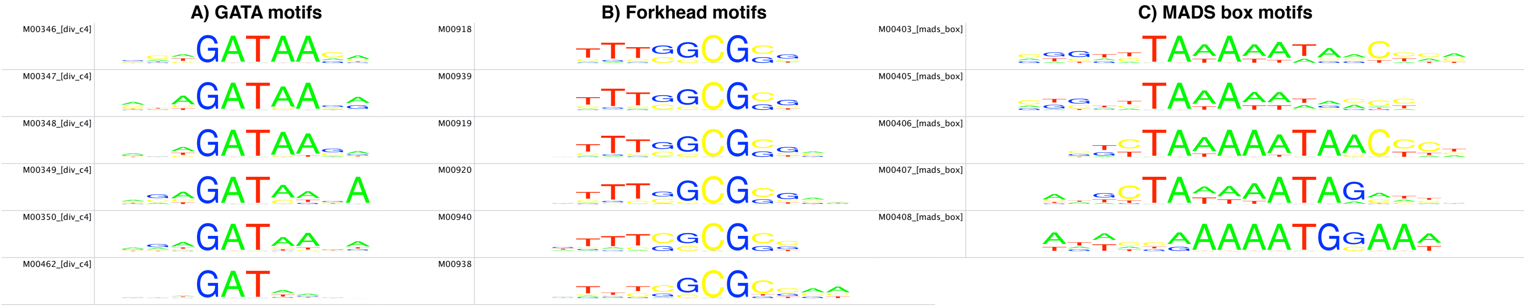
A) GATA motifs
B) Forkhead motifs
C) MADS box motifs
M00346_[div_c4]
M00347_[div_c4]
M00348_[div_c4]
M00349_[div_c4]
M00350_[div_c4]
M00462_[div_c4]
M00918
M00939
M00919
M00920
M00940
M00938
M00403_[mads_box]
M00405_[mads_box]
M00406_[mads_box]
M00407_[mads_box]
M00408_[mads_box]

Figure S3.

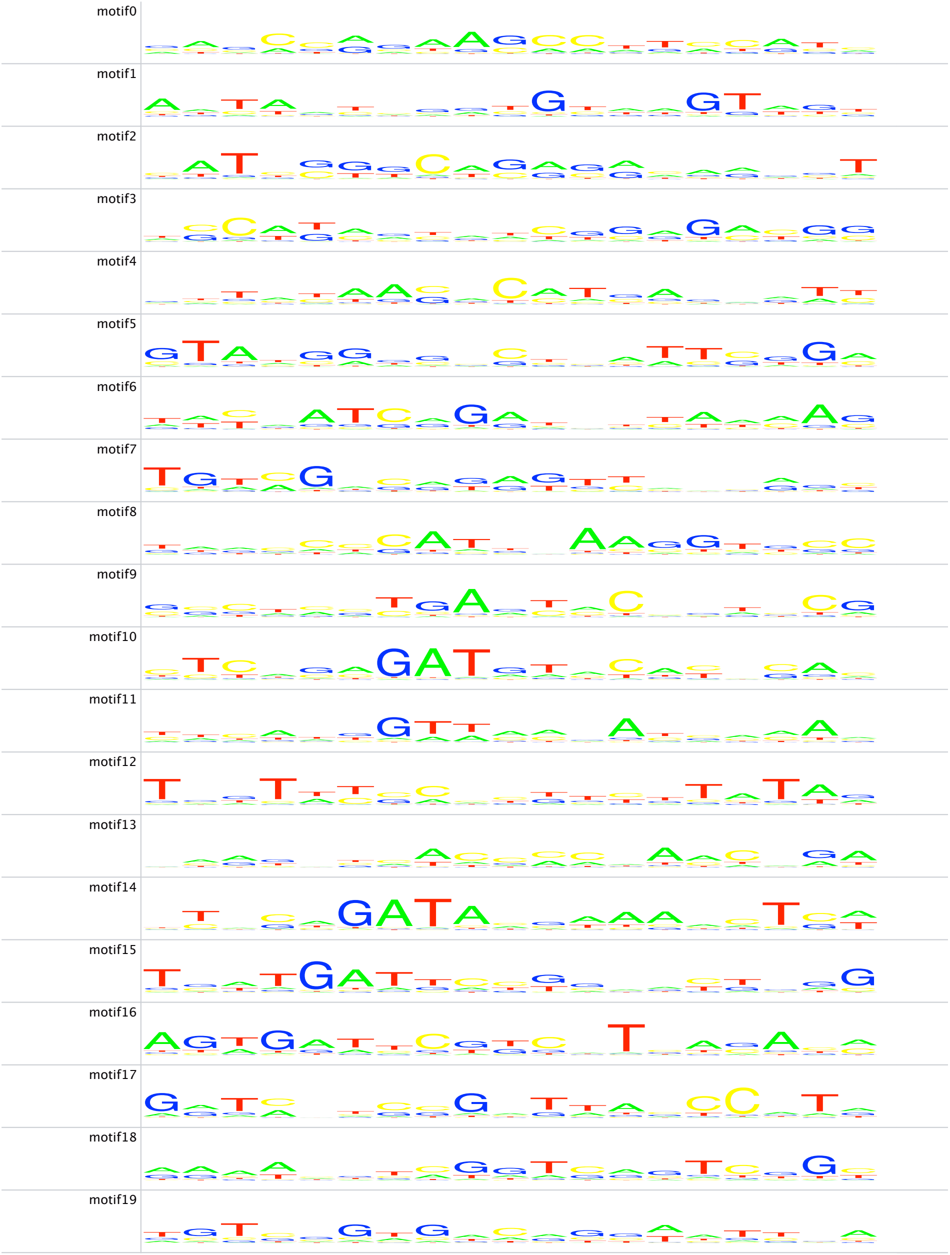
motif0
motif1
motif2
motif3
motif4
motif5
motif6
motif7
motif8
motif9
motif10
motif11
motif12
motif13
motif14
motif15
motif16
motif17
motif18
motif19

Figure S4A.

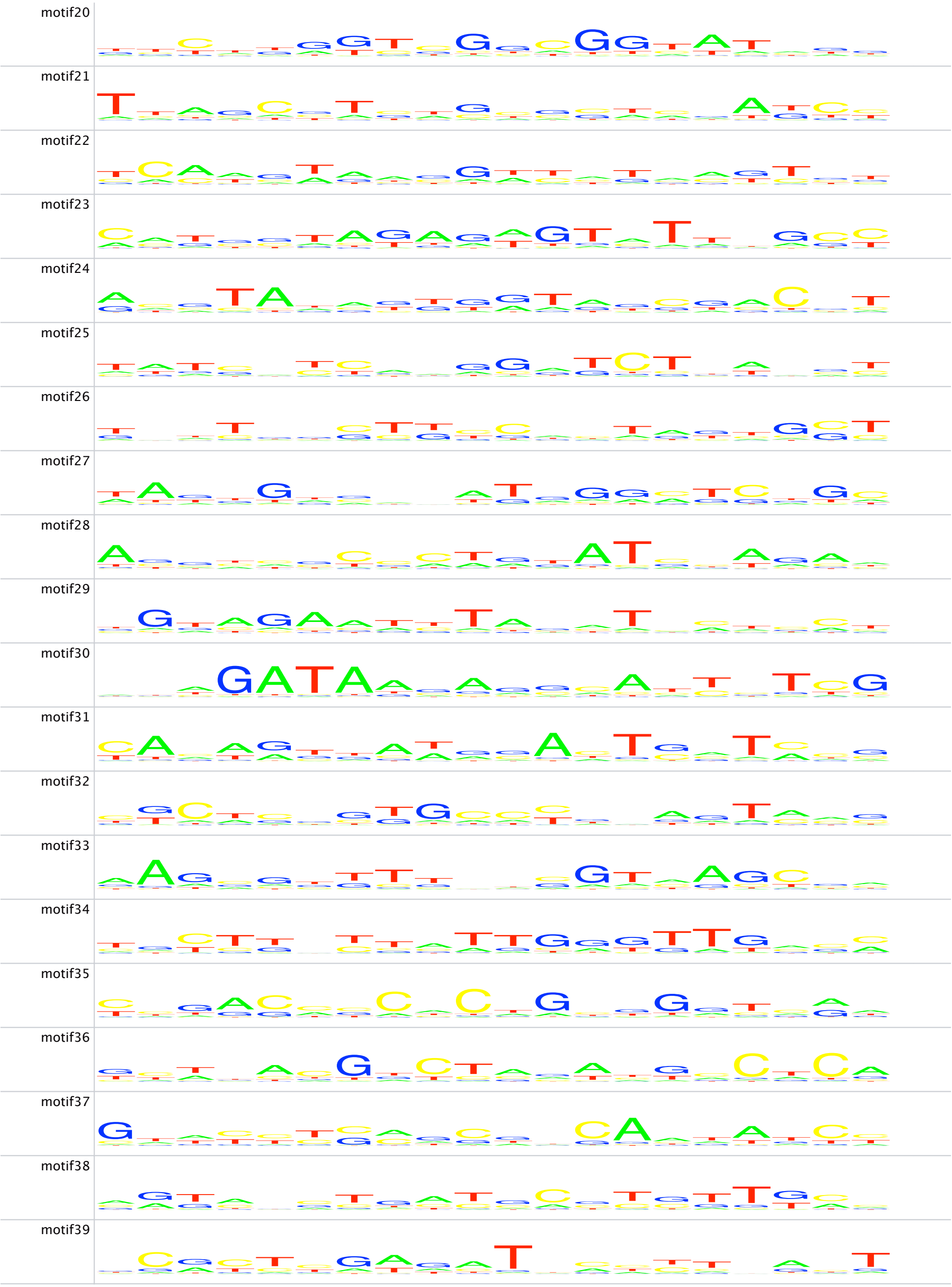
motif20
motif21
motif22
motif23
motif24
motif25
motif26
motif27
motif28
motif29
motif30
motif31
motif32
motif33
motif34
motif35
motif36
motif37
motif38
motif39

Figure S4B.

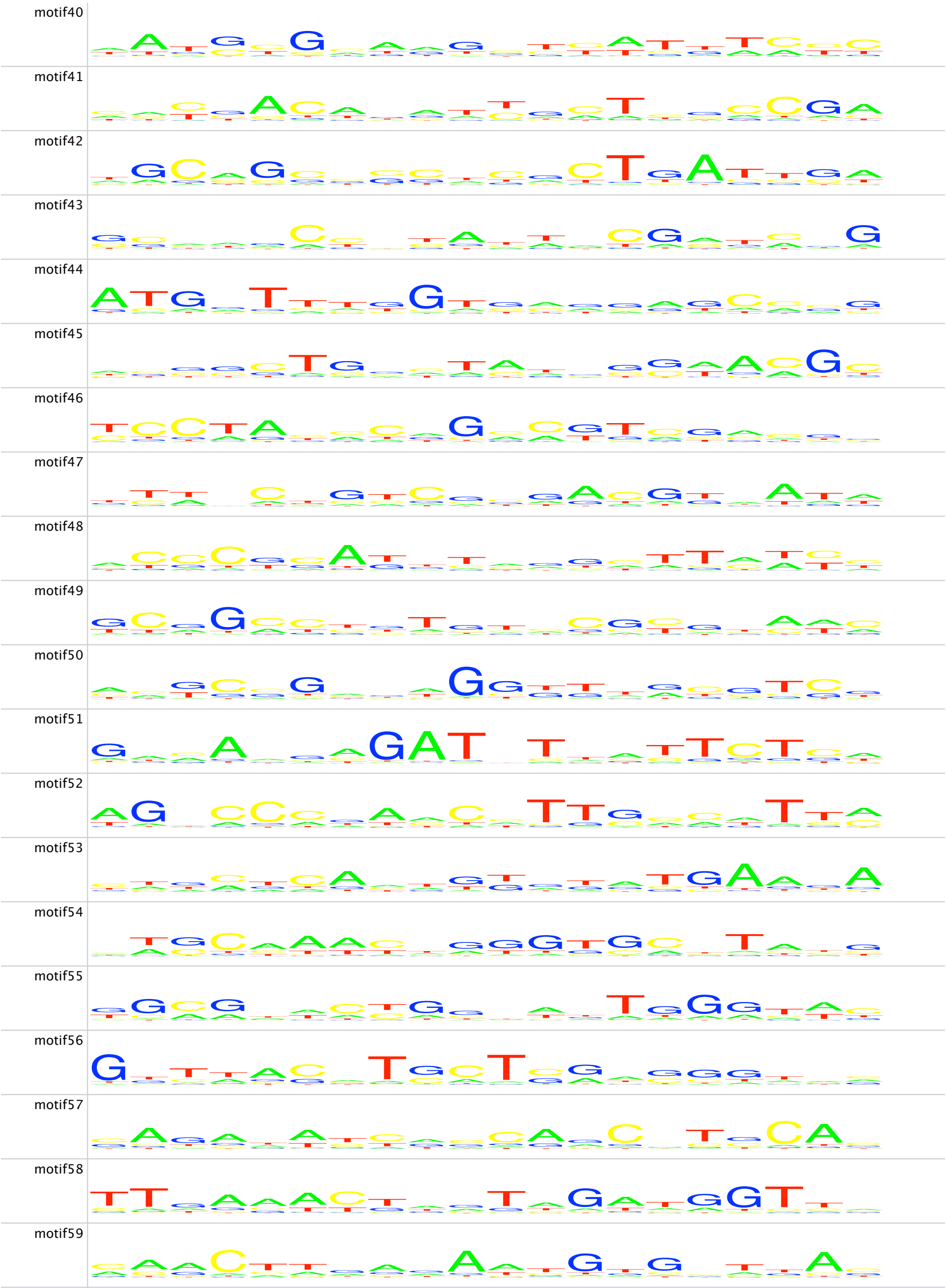
motif40
motif41
motif42
motif43
motif44
motif45
motif46
motif47
motif48
motif49
motif50
motif51
motif52
motif53
motif54
motif55
motif56
motif57
motif58
motif59

Figure S4C.

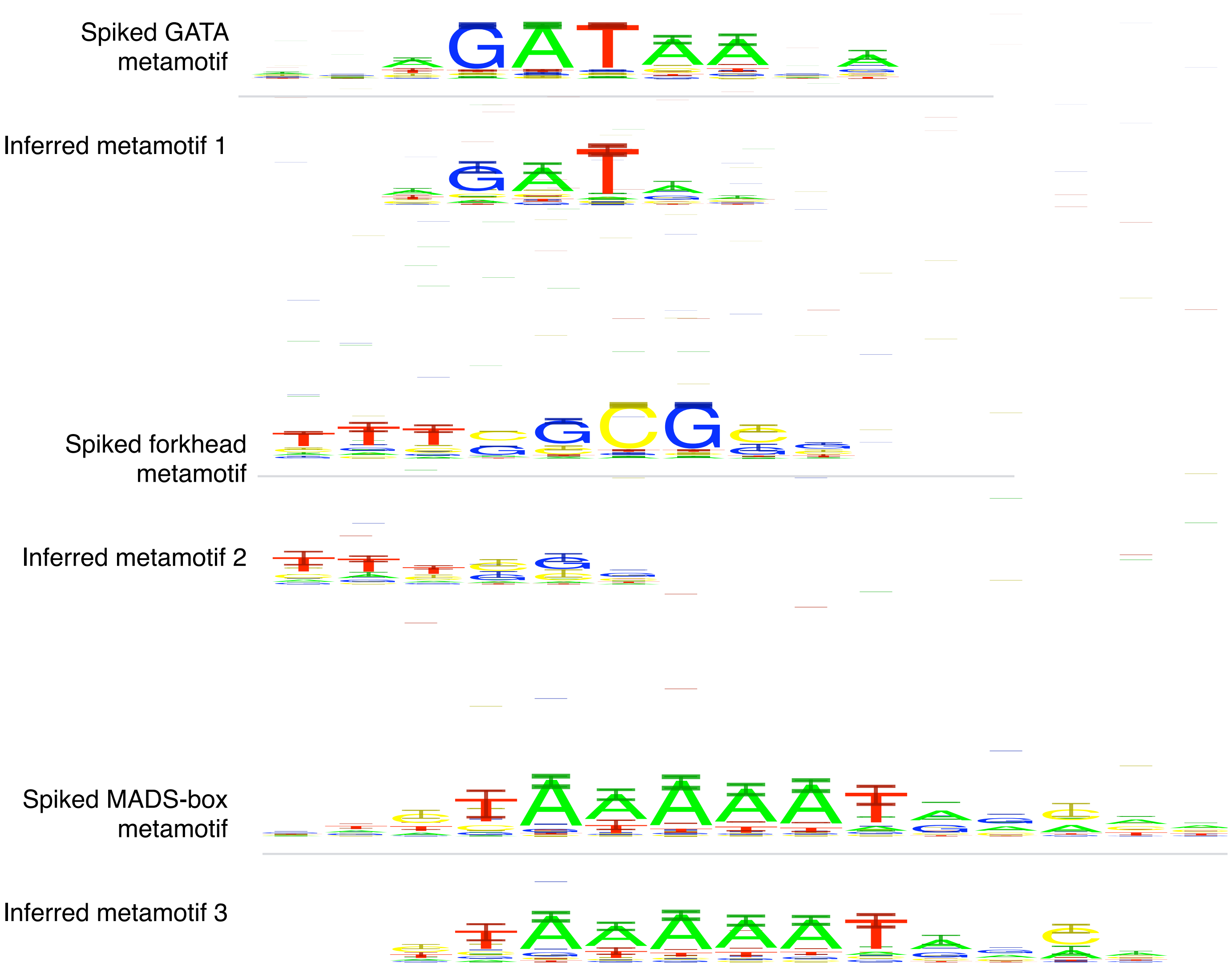
Spiked GATA metamotif
Inferred metamotif 1
Spiked forkhead metamotif
Inferred metamotif 2
Spiked MADS-box metamotif
Inferred metamotif 3

Figure S5.

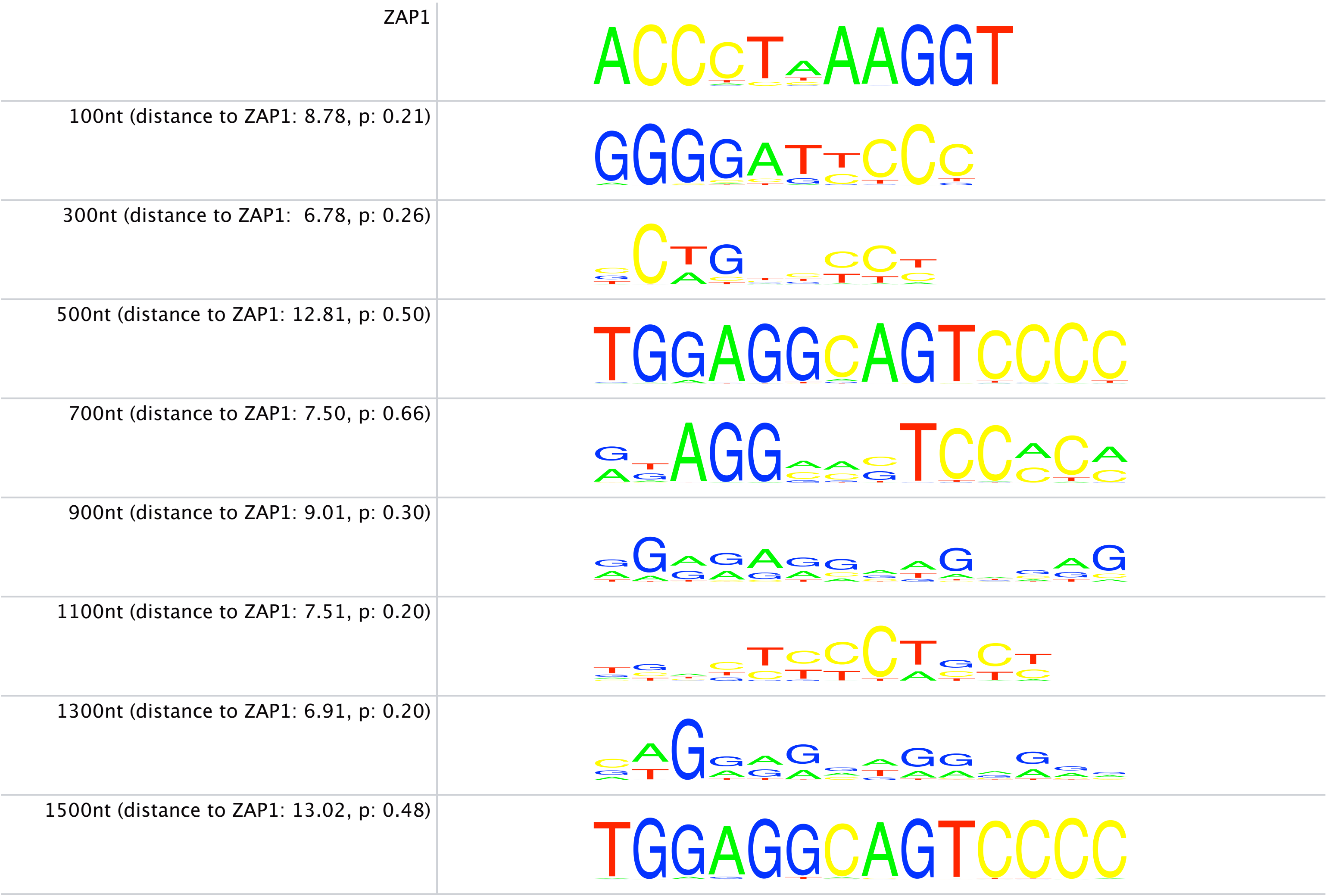
ZAP1
100nt (distance to ZAP1: 8.78, p: 0.21)
300nt (distance to ZAP1: 6.78, p: 0.26)
500nt (distance to ZAP1: 12.81, p: 0.50)
700nt (distance to ZAP1: 7.50, p: 0.66)
900nt (distance to ZAP1: 9.01, p: 0.30)
1100nt (distance to ZAP1: 7.51, p: 0.20)
1300nt (distance to ZAP1: 6.91, p: 0.20)
1500nt (distance to ZAP1: 13.02, p: 0.48)

Figure S6.

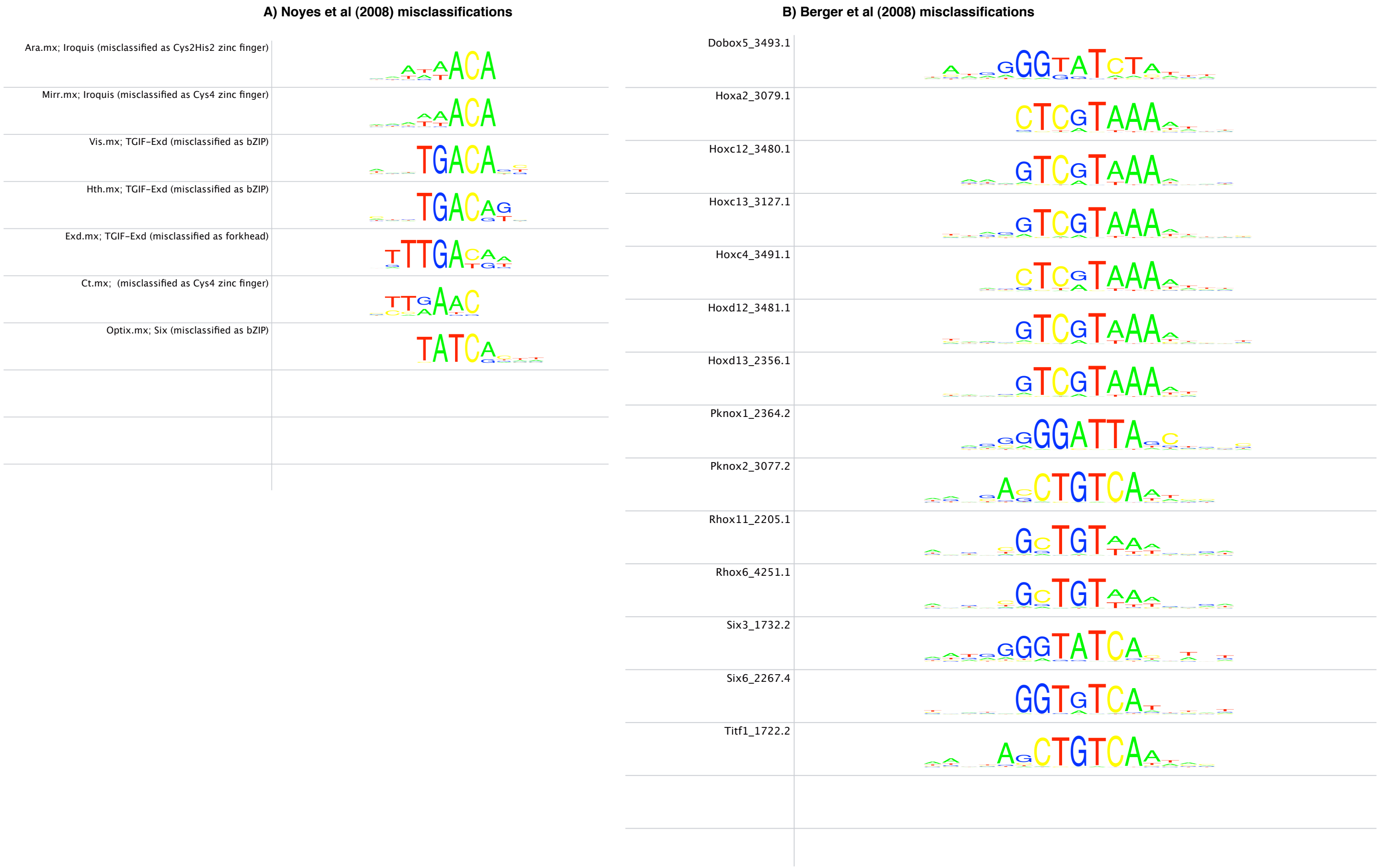
A) Noyes et al (2008) misclassifications
Ara.mx; Iroquis (misclassified as Cys2His2 zinc finger)
Mirr.mx; Iroquis (misclassified as Cys4 zinc finger)
Vis.mx; TGIF-Exd (misclassified as bZIP)
Hth.mx; TGIF-Exd (misclassified as bZIP)
Exd.mx; TGIF-Exd (misclassified as forkhead)
Ct.mx; (misclassified as Cys4 zinc finger)
Optix.mx; Six (misclassified as bZIP)
B) Berger et al (2008) misclassifications
Dobox5_3493.1
Hoxa2_3079.1
Hoxc12_3480.1
Hoxc13_3127.1
Hoxc4_3491.1
Hoxd12_3481.1
Hoxd13_2356.1
Pknox1_2364.2
Pknox2_3077.2
Rhox11_2205.1
Rhox6_4251.1
Six3_1732.2
Six6_2267.4
Titf1_1722.2

Figure S7.
